# Isolation of sesquiterpenoids from *Matricaria chamomilla* by means of solvent assisted flavor evaporation and centrifugal partition chromatography

**DOI:** 10.1007/s00216-021-03400-w

**Published:** 2021-05-28

**Authors:** Benedikt Slavik, Simon Roehrer, Helene M. Loos, Mirjana Minceva, Andrea Buettner

**Affiliations:** 1 Chair of Aroma and Smell Research, Friedrich-Alexander-Universität Erlangen-Nürnberg (FAU), Henkestr. 9, 91054 Erlangen, Germany; 2 Biothermodynamics, TUM School of Life Sciences, Technical University of Munich, Maximus-von-Imhof-Forum 2, 85354 Freising, Germany; 3 Fraunhofer Institute for Process Engineering and Packaging IVV, Giggenhauser Straße 35, 85354 Freising, Germany

**Keywords:** Sesquiterpenes, Countercurrent chromatography, Natural product, Bioactivity, Enrichment, Purification

## Abstract

The (semi)volatile fraction of *Matricaria chamomilla* L., an annual herbal plant from the family of Asteraceae, contains high quantities of sesquiterpenes and sesquiterpenoids. A method was developed to achieve isolation and separation of these compounds, using a combination of solvent assisted flavor evaporation (SAFE) and solid support-free liquid-liquid chromatography. The biphasic liquid solvent system *n*-heptane/ethyl acetate/methanol/water, 5/2/5/2 v/v/v/v (Arizona S) was elaborated as a suitable solvent system for the simultaneous separation of the target compounds. The lab-scale liquid-liquid chromatography separation performed in a countercurrent chromatography (CCC) column was successfully transferred to a semi-preparative centrifugal partition chromatography (CPC) column, which enabled the isolation of artemisia ketone, artemisia alcohol, α-bisabolone oxide A, and (*E*)-en-yn-dicycloether. α-Bisabolol oxide A and (*Z*)-en-yn-dicycloether co-eluted, but were successfully separated by subsequent size-exclusion chromatography (SEC). Similarly, spathulenol and α-bisabolol oxide B were obtained as a mixture, and were separated by means of column chromatography using silica gel as stationary phase. The isolated compounds were characterized by means of nuclear magnetic resonance spectroscopy (NMR) and gas chromatography–mass spectrometry (GC-MS).

**Supplementary Information:**

The online version contains supplementary material available at 10.1007/s00216-021-03400-w.

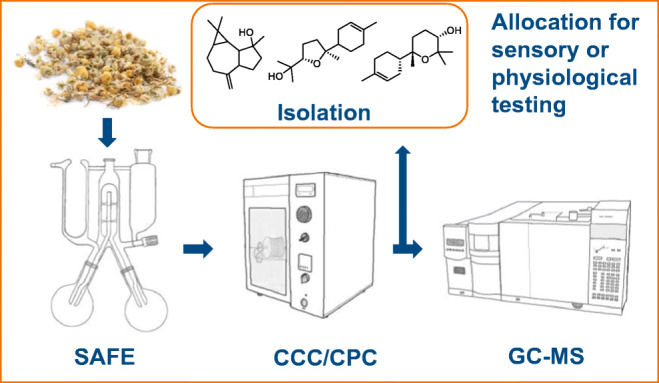

## Introduction

Sesquiterpenes and their derivatives, the sesquiterpenoids, occur in high amounts in some essential oils and contribute to a range of bioactive effects in humans such as anti-inflammatory, antiviral, or antioxidant activities [1]. They can also strongly influence the odor of these oils [2]. To characterize sesquiterpenoids in terms of their odor quality or their further physiological function, it is crucial to apply them in the highest purity to the respective biological test systems. Yet, the synthesis of sesquiterpenes and sesquiterpenoids is, due to their complex and diverse structures, very demanding. Accordingly, only few of these compounds are accessible, and alternative strategies need to be developed to obtain standards in the required purity. Besides biosynthetic approaches, which are of great interest especially with regard to stereoselective syntheses [3, 4], top-down approaches can provide an alternative route to obtain access to sesquiterpenes and sesquiterpenoids. Chromatographic methods, for instance the solid support-free liquid-liquid chromatography, better known as countercurrent chromatography (CCC), and centrifugal partition chromatography (CPC), can be used for the isolation of naturally occurring compounds [5–7]. In CCC/CPC, the phases of a multi-solvent biphasic liquid system are used, one as the stationary and the other one as the mobile phase, to separate a liquid mixture according to the specific distribution of the solutes between the two phases. A review of the role of this separation technology in Chinese herbal medicine research revealed that 12.6% of all isolated compounds belong to the general group of terpenes, comprising especially diterpenes and sesquiterpene lactones, with the other groups being mainly flavonoids (25.0%), polyphenols (15.7%), and alkaloids (15.4%) [8]. Sesquiterpenes and their derivatives have been, to date, less frequently investigated [8, 9]. Recently, we showed the applicability of CPC for the selective isolation of terpenoid structures from biotechnological fermentation [10, 11]. In addition, we successfully isolated the sesquiterpenoids rotundone and mustakone from *Boswellia sacra* volatile oil using CCC [2]. This approach thereby proved to be a suitable tool for the isolation of volatile as well as odor-active sesquiterpenoids.

In several current collaborative projects, we aim to characterize sesquiterpenes and sesquiterpenoids in relation to their sensory properties and their modulating activity on the human γ-aminobutyric acid (GABA_A_) receptor [12]. Apart from that, purified compounds are required to further build up our constantly growing substance library, comprising about 1400 substances, aiding us at establishing a comprehensive database on retention indices and mass spectrometric data, as well as human-sensory information such as odor qualities and odor thresholds in different matrices. Such data are the basis for digital representation and interpretation of analytical and sensory data, linking analytical and chromatographic information on specific molecular features and structural moieties with the percept elicited in humans. With respect to sesquiterpenoid substances as one specific substance class, we are interested in determining their contribution to the specific odors of several plant species, currently focusing, amongst others, on chamomile.

*Matricaria chamomilla* L. or *Matricaria recutita* L., also known as German chamomile, is an annual plant from the family of Asteraceae and has been used in traditional medicine since the early phases of humankind’s cultural development [13]. Main applications are the treatment of seizures or gastrointestinal inflammations, but this herbal remedy is also used externally, for instance in compresses for wound healing. The volatile fraction of the essential oil of *Matricaria chamomilla* L. is especially rich in sesquiterpenes and sesquiterpenoids [13–17]. Accordingly, chamomile was chosen to develop a method for the isolation and separation of the sesquiterpenoid fraction from other matrix-derived (semi)volatile constituents.

In the current study, we specifically aimed at developing a combined and consecutive protocol comprising a combination of different isolation and separation steps as a strategy to obtain the target molecules at the highest purity. Solvent assisted flavor evaporation (SAFE) was chosen as the first mild isolation step as the volatile fraction is obtained as solvent distillate at moderate temperatures with distillation under high vacuum [18]. The distillate obtained from SAFE was then separated by solid support-free liquid-liquid chromatography (CCC/CPC). Further purification steps included column chromatography techniques using silica gel as stationary phase with different solvents and solvent mixtures as mobile phases. Additionally, size-exclusion-chromatography (SEC) using Sephadex™ LH-20 as column material was applied to achieve complete separation of the target sesquiterpenoids. Our main goal was to obtain several compounds from chamomile in the highest purity. Therefore, only the final yields and amounts were determined. For future investigations, it might be interesting to monitor the yields and amounts of the different isolation steps. This will allow, on the one hand, to compare the here presented isolation strategy with alternative strategies such as preparative gas chromatography, and on the other hand provide insights into potential optimization parameters.

## Materials and methods

### Chemicals

All solvents were either purified by distillation or used in LC-MS grade. Dichloromethane (DCM), ethanol, ethyl acetate, *n*-heptane, *n*-hexane, methanol, and sodium sulfate (anhydrous) were purchased from VWR (VWR International, Radnor, PA, USA). Acetonitrile, toluene, and the series of *n*-alkanes (C_6_-C_26_) were purchased from Sigma-Aldrich (Sigma-Aldrich Corporation, St. Louis, MO, USA). *tert*-Butyl methyl ether was obtained from Thermo Fisher (Thermo Fisher Scientific, Waltham, MA, USA), and trichloromethane D1 (0.03% v/v) from Carl Roth (Carl Roth GmbH + Co. KG, Karlsruhe, Germany). Ultrapure water was used for the experiments (Thermo Fisher Scientific, Waltham, MA, USA). For the CCC and CPC experiments, the solvents *n*-heptane, ethyl acetate, and methanol of the biphasic solvent system *n*-heptane/ethyl acetate/methanol/water (Arizona S) were all from Merck (Merck KGaA, Darmstadt, Germany) in analytical grade (purity ≥99%). Milli-Q water was obtained from a Milli-Q Direct Water Purification System (Merck Millipore, Billerica, MA, USA).

### Solvent extraction and solvent assisted flavor evaporation (SAFE)

Dried chamomile flower heads, cultivated in Latvia, were purchased from a local store in Erlangen, Germany. A total amount of 250 g dried flower heads were finely chopped (Kenwood mini chopper CH180, 300 W, Kenwood Limited, Havant, UK) until a yellowish powder was obtained. After addition of DCM (2 L), the crude mixture was stirred for 2.5 h and subsequently filtered. The brownish solution was dried over sodium sulfate and was divided equally into four fractions, which were distilled at 65 °C under vacuum by means of the SAFE technique [18]. The distillates were reduced to ~1.2 mL by Vigreux and microdistillation at 50 °C [19].

### Gas chromatography–mass spectrometry (GC-MS)

Analyses of the chamomile distillate and the CCC/CPC samples were performed on a GC 6890 (Agilent Technologies, Santa Clara, CA, USA) connected to a MSD 5973 (Hewlett-Packard, Palo Alto, CA, USA), equipped with a GERSTEL MPS 2 multipurpose sampler and a GERSTEL CIS 3 injection system (both from GERSTEL GmbH & Co. KG, Mülheim an der Ruhr, Germany), which was run in split mode (5:1) using a baffled glass liner and an injection volume of 2 μL. A DB-5 capillary column was used (30 × 0.25 mm, film thickness 0.25 μm), connected to an uncoated fused silica capillary pre-column (3 m × 0.53 mm; both from Agilent J&W Scientific, Santa Clara, CA, USA). The temperature program was started at 40 °C or 100 °C (used for samples in MeOH), respectively, with a hold time of 1 min. The temperature was then raised by 8 °C/min until 300 °C and held for 3 min. Helium was used as carrier gas with a flow of 1.0 mL/min. EI mass spectra were recorded using 70 eV in full scan mode (40–400 amu). For identification, the mass spectra were compared with the NIST mass spectral library (version 2.0, National Institute of Standards and Technologies, Gaithersburg, MD, USA).

GC-MS analyses of the target compounds were performed on a GC 7890 connected to an MSD 5975C (both from Agilent Technologies, Santa Clara, CA, USA) equipped with a GERSTEL MPS 2 multipurpose sampler and a GERSTEL CIS 4 injection system (both from GERSTEL GmbH & Co. KG, Mülheim an der Ruhr, Germany). Samples were applied via the on-column technique (1 μL). Capillary columns were used, as described above. The temperature program was started at 40 °C with a hold time of 2 min. The temperature was then raised by 8 °C/min until 300 °C and held for 5 min. Helium was used as carrier gas with a flow of 1.0 mL/min. EI mass spectra were recorded using 70 eV in full scan mode (40–400 amu).

### Biphasic solvent system screening with predictive thermodynamic model

For the theoretical solvent system screening, the conductor-like screening model for real solvents (COSMO-RS) was used [20]. In this work, the software COSMOconfX (Version 4.0, COSMOlogic, Leverkusen, Germany) and COSMOthermX (Version: C30 Release 16.01., COSMOlogic, Leverkusen, Germany) was used. The approach used for the screening and selection of biphasic solvent systems for CCC/CPC separations is described in detail in the literature [21–23].

### Preparation of biphasic liquid systems

For the CCC/CPC separations the biphasic liquid system *n*-heptane/ethyl acetate/methanol/water, 5/2/5/2 v/v/v/v (Arizona S) was prepared by mixing the respective portions of the solvents at room temperature. The mixture was vigorously shaken and equilibrated at room temperature for at least 2 h. Prior to the CCC/CPC experiments, the phases were split, and placed in two separate storage vessels and degassed in an ultrasonic bath. A freshly prepared biphasic system was used in each experiment.

### Countercurrent chromatography (CCC)

The CCC experiments were carried out on a countercurrent chromatography column, model HPCCC-Mini Centrifuge (0.8 mm ID PTFE, β-value range between 0.5 and 0.78) from Dynamic Extractions (Tredegar, Wales), with a total column volume of 18.2 mL. The CCC-Mini was connected to a cooling unit (FL 300, Julabo, Seelbach, Germany) and two isocratic HPLC pumps (Gilson 306, Gilson, Middleton, WI, USA) for delivering the mobile and stationary phase. The effluent was monitored with a DAD 171 diode array detector (Gilson 171 UV-DAD-detector, Gilson, Middleton, WI, USA) at a wavelength of 280 nm. The CCC experiments were performed at room temperature in descending mode using the lower phase as the mobile phase. At the beginning of each experiment, the column was filled with the stationary phase (upper phase). Afterwards, the rotational speed was set to 1900 rpm, and the mobile phase was pumped through the column at 1 mL/min until no more stationary phase eluted from the column. A feed sample of 227 mg/mL (distillate dissolved in upper phase) was then introduced through a manual 6-port valve and an injection loop of 0.5 mL, while the mobile phase was continuously pumped at 1 mL/min. The CCC run was manually fractionated after injection in fraction intervals of 1 min. The collected fractions were diluted 1:1 with methanol, dried over sodium sulfate, and directly analyzed by GC-MS.

### Centrifugal partition chromatography (CPC)

The CPC experiments were performed on a SCPC-250 column (Armen Instrument, Saint Ave, France). The column consisted of two single columns connected in series with an experimentally determined total volume of 182 mL. The column was equipped with two HPLC gradient pumps (Armen Instrument, Saint Ave, France), one for filling the column with the stationary phase and the other one for pumping the mobile phase during the separation. The effluent was monitored with a UV detector (ECOM DAD600 2WL 200–600 nm, Prague, Czech Republic) at 280 nm. A fraction collector (LS 5600, Armen Instrument, Saint Ave, France) was used for collecting fractions during the separation run. The experimental procedure is equivalent to the procedure described for the CCC experiment. In CPC, the rotational speed was set to 1700 rpm and the flow rate to 12 mL/min. A feed sample of 261 mg/mL (distillate dissolved in upper phase) was introduced via a manual injection valve and an injection loop of 2 mL. The run was fractionated by a fraction collector in intervals of 1 min. The collected fractions were diluted 1:1 with methanol, dried over sodium sulfate, and subsequently analyzed by GC-MS. For the following separation steps, fractions containing either the single target compounds or a two-component mixture were combined. The solvents were removed under reduced pressure and the remaining aqueous phase was extracted twice with DCM. The combined organic phases were dried over sodium sulfate and the pure substances were obtained after removing the solvent under reduced pressure.

### Simulation of CPC separations

The simulations for the estimation of the separation method transferability were performed with the gPROMS Model Builder v4.2 software from Process Systems Enterprise (London, UK) numerically solving the equilibrium cell model equations. In this context, the CPC column is described as a cascade of *N* ideal stirred-tank reactors (theoretical stages) [24].

The stationary phase retention (*S*_*F*_) in the column describes the fraction of the column volume occupied by the stationary phase:

1 $$ {S}_F=\frac{V_{SP}}{V_C} $$

where *V*_*SP*_ is the volume of the stationary phase and *V*_*C*_ is the total column volume. The separation depends on the different distribution of the compounds between the stationary and mobile phase, which can be described by the different partition coefficients (*P*_*i*_) of the compounds:

2 $$ {P}_i=\frac{c_i^{SP}}{c_i^{MP}} $$

where ${c}_i^{SP}$ is the concentration of solute *i* in the stationary phase and ${c}_i^{MP}$ in the mobile phase.

For each component i, the mass balance in each theoretical stage is solved according to Eq. 1 assuming ideal mixing in each stage. Details about the model can be found in previous works [25–27].

3 $$ \frac{{\mathrm{V}}_{\mathrm{C}}\left(1-{\mathrm{S}}_{\mathrm{F}}\right)}{{\mathrm{N}}_{\mathrm{i}}}\frac{\mathrm{d}{\mathrm{c}}_{\mathrm{i},\mathrm{k}}^{\mathrm{MP}}}{\mathrm{d}\mathrm{t}}+\frac{{\mathrm{V}}_{\mathrm{C}}{\mathrm{S}}_{\mathrm{F}}}{{\mathrm{N}}_{\mathrm{i}}}\frac{{\mathrm{d}\mathrm{c}}_{\mathrm{i},\mathrm{k}}^{\mathrm{S}\mathrm{P}}}{\mathrm{d}\mathrm{t}}=\mathrm{F}\ \left({\mathrm{c}}_{\mathrm{i},\mathrm{k}-1}^{\mathrm{MP}}-{\mathrm{c}}_{\mathrm{i},\mathrm{k}}^{\mathrm{MP}}\right) $$

where cell k = 1, 2, …, N_i_ refers to the number of theoretical stages, ${\mathrm{c}}_{\mathrm{i},\mathrm{k}}^{\mathrm{MP}}$ is the concentration of compound i in the mobile phase in cell k, ${\mathrm{c}}_{\mathrm{i},\mathrm{k}}^{\mathrm{SP}}$ is the concentration of compound i in the stationary phase of cell k, and F is the total mobile phase flow rate. For simplicity, N_i_ = 400 was assumed as a constant value for all target compounds, which was estimated as a minimum column efficiency based on previous studies under similar conditions [28]. Since the absolute concentration of the compounds in the feed was unknown, the relative concentration of each compound based on the peak area from the GC-MS analysis was used for the simulation.

### Size-exclusion chromatography (SEC)

CPC fractions containing a mixture of **7** and **8** were combined and diluted with methanol and were subsequently applied on a Sephadex™ LH-20 column (GE Healthcare Bio-Sciences AB, Uppsala, Sweden). Methanol was chosen as eluent, the fractions were collected manually. After the combination of fractions with sufficient purity (based on GC-MS measurements), methanol was removed under reduced pressure.

### Column chromatography

CPC fractions containing a mixture of **4** and **5** were combined, dissolved in the mobile phase (*n*-hexane/ethyl acetate (3:1)), and subsequently applied for consecutive silica gel column chromatography (silica gel 40–63 μm, VWR International, Radnor, PA, USA). The eluents were chosen in the following sequence. *n*-Hexane/ethyl acetate (3:1), *n*-hexane/ethyl acetate (7:1), and toluene/MTBE (3:1) which was conducted twice.

### GC-MS and NMR

GC-MS and NMR spectra were recorded to determine the purity of the isolated compounds.

Artemisia ketone (**1**): EI-MS: 83 (100), 55 (25), 41 (7), 84 (5), 40 (5), 53 (5), 69 (3), 67 (2), 51 (1), 43 (1)

Artemisia alcohol (**2**): EI-MS: 85 (100), 41 (21), 55 (11), 67 (9), 93 (8), 43 (7), 70 (7), 40 (7), 91 (6), 79 (6)

(*E*)-β-farnesene (**3**): EI-MS: 69 (100), 93 (64), 41 (48), 133 (27), 79 (25), 67 (24), 81 (21), 91 (20), 120 (19), 55 (15)

Spathulenol (**4**): Yellowish, viscous liquid, 4.93 mg; EI-MS: 43 (100), 91 (82), 205 (81), 41 (69), 119 (68), 93 (58), 105 (57), 79 (54), 159 (46), 107 (44)

α-Bisabolol oxide B (**5**): Yellowish, viscous liquid, 21.35 mg; EI-MS: 143 (100), 43 (84), 105 (78), 85 (64), 59 (61), 81 (60), 161 (55), 93 (49), 41 (44), 71 (42)

α-Bisabolone oxide A (**6**): Colorless, viscous liquid, 5.1 mg; EI-MS: 93 (100), 141 (80), 43 (73), 94 (61), 67 (54), 121 (49), 95 (47), 68 (41), 79 (38), 41 (36)

α-Bisabolol oxide A (**7**): Colorless, viscous liquid, 6.0 mg; EI-MS: 143 (100), 43 (60), 93 (38), 125 (35), 67 (29), 71 (27), 107 (27), 68 (26), 121 (23), 59 (22); 1H NMR (CDCl3, 600 MHz) δH 1.14/1.20/1.29 ppm (3x3H, s, CH3), 2.03-1.23 (11H, br m, 5xCH2, 1xCH), 3.46 (dd, J=6.0, 3.0 Hz, CH-OH), 5.39 (1H, s, C=CH)

(*Z*)-en-yn-dicycloether (**8**): Yellowish, viscous liquid, 5.37 mg; EI-MS: 200 (100), 115 (59), 128 (37), 76 (34), 129 (31), 157 (29), 199 (27), 170 (27), 102 (27), 50 (27)

(*E*)-en-yn-dicycloether (**9**): Yellowish, viscous liquid, 4.6 mg; EI-MS: 200 (100), 115 (57), 128 (37), 76 (34), 129 (30), 157 (29), 102 (26), 50 (26), 199 (26), 170 (26); 1H NMR (CDCl3, 600 MHz, impurity 8, attribution to the respective isomer was not always unambiguous) δH 1.60 ppm (3H, s, CH3), 2.30-2.00 (4H, m, O-CH2-CH2-CH2-C), 4.04-3.99/4.28-4.18 (2H, m, O-CH2), 4.95(*E*)/4.62(*Z*) (1H, s, =CH≡), 6.18(*Z*)/6.23(*E*) (1H, d/dd J= 5.7/5.7, 1.7 Hz, C-CH2=CH2-C=), 6.26(*Z*)/6.72(*E*) (1H, d/d J= 5.7/5.7, C-CH2=CH2-C=)

See Supplementary Information (ESM) for NMR

## Results and discussion

### Extraction and enrichment of sesquiterpenes and sesquiterpenoids

**Fig. 1:**
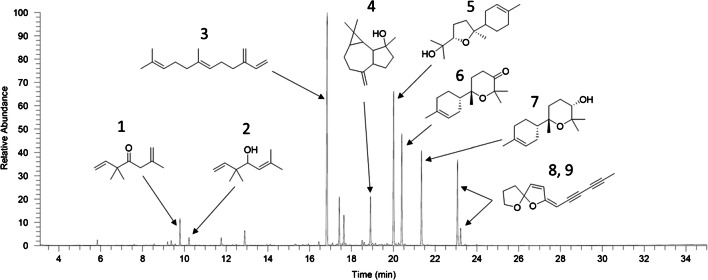
Total ion chromatogram of the chamomile distillate obtained after SAFE distillation (DB-5 column, injection of 2 μL in split mode (5:1), 40 °C for 1 min, 8 °C/min until 300 °C, hold time 3 min, electron impact mode (EI), 70 eV); **1** = artemisia ketone, **2** = artemisia alcohol, **3** = (*E*)-β-farnesene, **4** = spathulenol, **5** = α-bisabolol oxide B, **6** = α-bisabolone oxide A, **7** = α-bisabolol oxide A, **8**/**9** = (*Z*/*E*)-en-yn-dicycloether

The chemical composition of the chamomile distillate after SAFE was investigated by means of GC-MS. A representative chromatogram is shown in Fig. 1. The volatile fraction mainly consisted of sesquiterpenes and sesquiterpenoids eluting at retention times ranging from around 15.5 to 22.5 min, which corresponds to retention indices (RI) 1375–1850. By comparison with mass spectra and RIs in the literature [29] and the NIST mass spectral library, major substances were tentatively identified as (*E*)-β-farnesene (16.84 min, RI 1456, **3**), spathulenol (18.90 min, RI 1578, **4**), α-bisabolol oxide B (20.01, 1667, **5**), α-bisabolone oxide A (20.41, 1694, **6**), and α-bisabolol oxide A (21.35, 1763, **7**). The peaks detected at 23.07 min (RI 1894, **8**) and 23.22 min (RI 1906, **9**) correspond to the (*Z*)- and (*E*)-isomers of en-yn-dicycloether ((*E*/*Z*)-2-(hexa-2,4-diyn-1-ylidene)-1,6-dioxaspiro[4.4]non-3-ene). According to the literature the (*Z*)-isomer elutes on a DB-5 column before the (*E*)-isomer [29]. Minor compounds were artemisia ketone (9.79 min, RI 1059, **1**) and artemisia alcohol (10.22 min, RI 1081, **2**). Other minor compounds were not further analyzed. The composition of the volatile fraction of chamomile is in good agreement with previous reports [13–17]. The SAFE technique is an easy and efficient tool to isolate volatile substances from matrix material, which, however, discriminates substances with higher boiling points [18]. To reduce discrimination, the distillation temperature was increased to 65 °C. Still, GC-MS measurements indicated that some higher boiling sesquiterpenes and sesquiterpenoids were present in the residue. The yield may be increased by raising the temperature and by re-using the residue for repetitive distillation with higher amounts of solvent. Besides using this distillate, for future investigations, it might also be interesting to consider chamomile essential oils as a starting material, since the composition is similar to the SAFE distillate. Nevertheless, the quantity of sesquiterpenes and sesquiterpenoids in the distillate was, on the basis of GC-MS measurements, judged to be sufficient to proceed with further separation steps, using CCC/CPC.

### Screening and selection of the biphasic solvent system for CCC/CPC

Due to the wide variety of possible biphasic liquid solvent systems, the most challenging step of the separation method development in CCC/CPC is the selection of a suitable biphasic liquid system. In this study, a thermodynamic model–based prediction approach was used to avoid time-consuming experimental effort, e.g., by performing trial-and-error shake-flask experiments. The scope of the study was to find a biphasic solvent system that allows the simultaneous separation of the major compounds in the chamomile distillate. The conductor-like screening model for real solvents (COSMO-RS) was used as suggested in previous works to screen biphasic solvent systems [20–23]. In the biphasic liquid system, the target compounds should have a partition coefficient (P_i_) in, or close to, the preferred “sweet spot” range (0.4 < P_i_ < 2.5), as well as a separation factor greater than 1.5. The “sweet spot” range provides a good compromise between the separation resolution, productivity, and solvent consumption to achieve a sufficient separation in CCC/CPC [30]. Based on the molecular structure of the solvents and chamomile compounds, the partition coefficients were predicted in several biphasic solvent systems. Covering a broad polarity range, different compositions of systems commonly used in CCC/CPC were screened, including *n*-heptane/ethyl acetate/methanol/water (predefined compositions also known as Arizona series), *n*-hexane/ethyl acetate/methanol/water (predefined compositions also known as HEMWat series), butanol/methanol/water, *n*-hexane/ethyl acetate/acetonitrile, and *n*-hexane/ethanol/acetonitrile. The system *n*-heptane/ethyl acetate/methanol/water, 5/2/5/2 v/v/v/v (known as Arizona S), and *n*-hexane/ethyl acetate/methanol/water, 1/1/1/1 v/v/v/v (known as HEMWat 0), turned out to be potentially most promising for a separation of components **1**–**9**. Due to the general demand for the substitution of *n*-hexane with alternatives like cyclohexane or *n*-heptane, the Arizona S system was selected for the CCC separation.

### Lab-scale CCC experiments and transfer to a semi-preparative CPC column

**Fig. 2:**
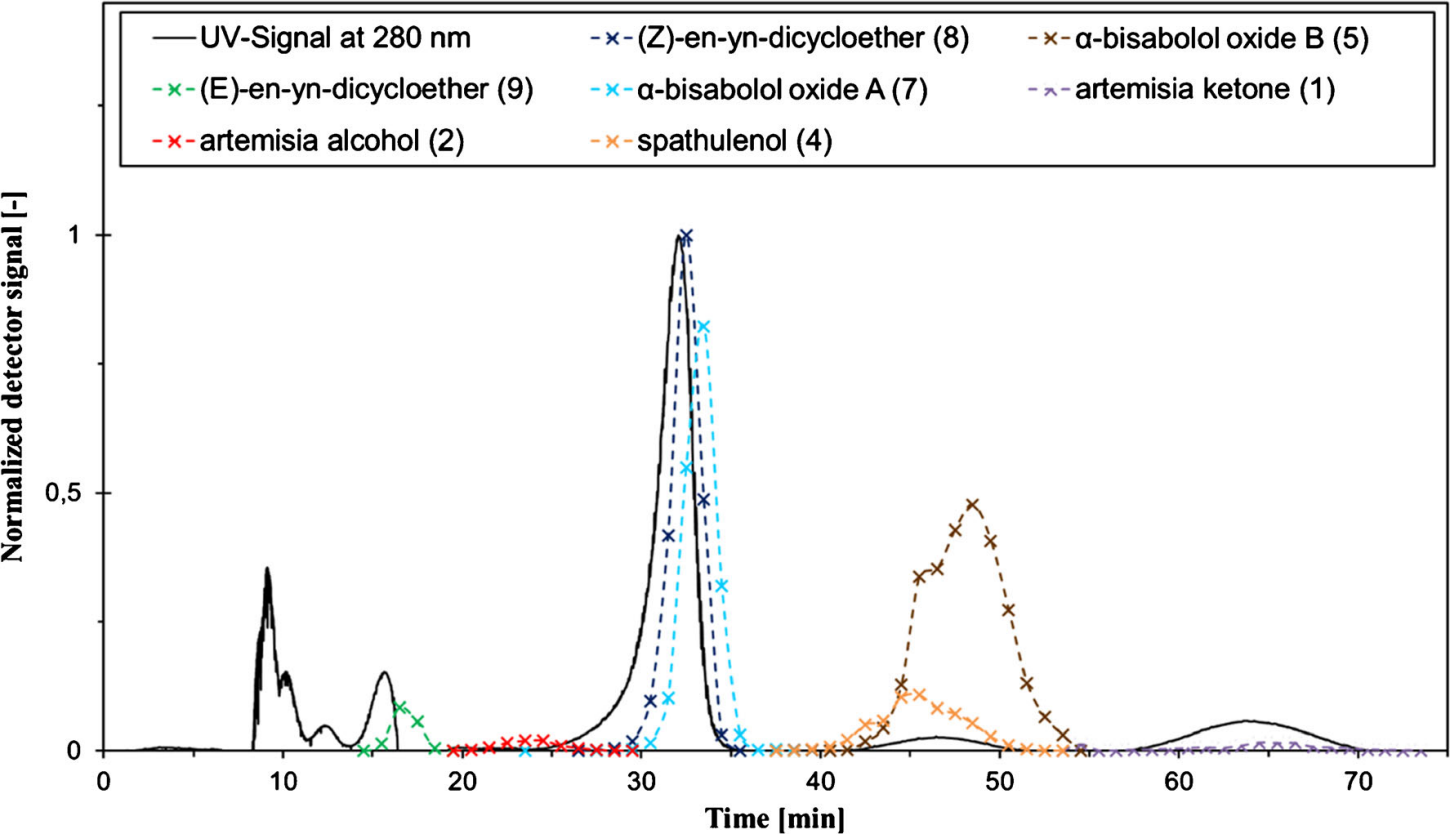
Chromatogram of the CCC separation of the chamomile distillate, using the solvent system Arizona S (*n*-heptane/ethyl acetate/methanol/water 5/2/5/2 v/v/v/v) at a wavelength of 280 nm, including the GC-MS offline analysis of the collected fractions. DSC mode: 1 mL/min, c_inj_ = 227 mg/mL dissolved in UP, V_inj_ = 0.5 mL, 1900 rpm, S_F_ = 0.63

The selected biphasic solvent system was first tested in a small lab-scale CCC unit in descending mode (DSC) in which the lower phase of the biphasic system is used as the mobile phase. The feed sample was prepared by dissolving the chamomile distillate in the upper phase (UP), which was used as the stationary phase. This was necessary to achieve a high column load, since the distillate, which was prepared with DCM as the solvent, was poorly soluble in the aqueous lower phase of the Arizona S system. With this approach, a total column load of 113.5 mg chamomile distillate (c_feed_ = 227 mg/mL, V_inj_ = 0.5 mL) could be applied. After the sample injection, no stationary phase loss was observed over the entire runtime. Fractions were collected every 60 s and were analyzed by GC-MS. In Fig. 2, the reconstructed CCC chromatogram derived from GC-MS offline analysis of the collected fractions is shown. The compounds artemisia alcohol (**2**), (*E*)-en-yn-dicycloether (**9**) and artemisia ketone (**1**) were baseline separated in pure fractions, while co-elution occurred between spathulenol (**4**) and α-bisabolol oxide B (**5**) as well as α-bisabolol oxide A (**7**) and (*Z*)-en-yn-dicycloether (**8**). Furthermore, α-bisabolone oxide A (**6**) was detected at the end of the run, which is however not depicted in Fig. 2. The results of the CCC pulse injection experiment at lab-scale showed a sufficient separation of the target compounds. Subsequently, this was transferred to a semi-preparative CPC column (182 mL total column volume). First, the transferability of the separation method from the lab-scale CCC to the semi-preparative CPC was estimated due to limited availability of the raw extract and in order to avoid various trial-and-error experiments. The elution profile was first simulated using the cell model to confirm that sufficient separation is possible for selected operating conditions [24]. The mobile phase flow rate of 12 mL/min was selected in order to achieve the same stationary phase retention as in the lab-scale CCC separation (S_F_ = 0.63 in CCC). In addition, a similar feed concentration (c_inj_ = 261 mg/mL) of the chamomile distillate dissolved in the upper phase (stationary phase) was used in order to prevent possible phase destabilization in the column due to surface-active compounds potentially being present in the extract. It was found that the injection volume could be increased up to 2 mL to still achieve a full separation between compound **2** and the binary mixture of **7**/**8**. In the simulation study, the P_i_-values of the target compounds calculated from the CCC offline chromatogram (Fig. 2) were used: artemisia ketone (**1**) = 5.0; artemisia alcohol (**2**) = 1.5; spathulenol (**4**) = 3.4; α-bisabolol oxide B (**5**) = 3.6; α-bisabolol oxide A (**7**) = 2.3; (Z)-en-yn-dicycloether (**8**) = 2.2; (*E*)-en-yn-dicycloether (**9**) = 0.9.

**Fig. 3:**
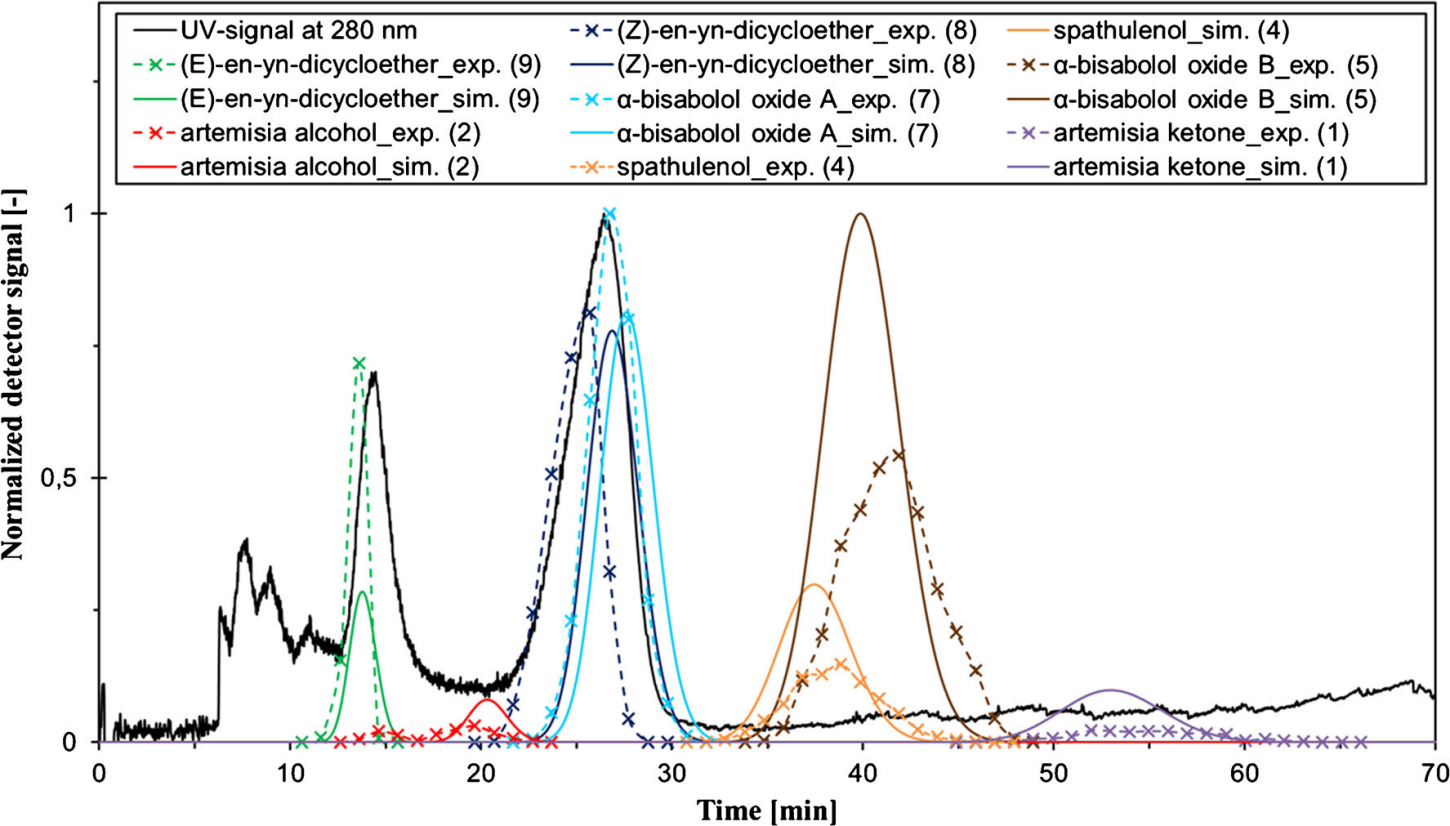
Chromatogram of the CPC separation of the chamomile distillate with the solvent system Arizona S (*n*-heptane/ethyl acetate/methanol/water 5/2/5/2 v/v/v/v) at a wavelength of 280 nm (black line), including the GC-MS offline analysis of the collected fractions obtained by the CPC separation (dashed colored lines) as well as the simulated chromatogram (continuous colored lines). DSC mode: 12 mL/min, c_inj_ = 261 mg/mL dissolved in UP, V_inj_ = 2 mL, 1700 rpm, S_F_ = 0.62

However, a slight but constant stationary phase loss was observed during the whole experimental run. This was most likely due to the different column geometry of the used CPC in comparison to the CCC column. The fact that the stationary phase was introduced with the feed as well as the chamomile distillate potentially containing surface-active compounds can additionally destabilize the biphasic system in such columns. As apparent from Fig. 3, the simulated chromatogram and the offline chromatogram of the CPC separation agree considerably well. Hence, the separation was successfully transferred from CCC to CPC, resulting in pure fractions of **1**, **2**, **6**, and **9** and binary mixtures of **7** and **8**, and **4** and **5**.

In both separations, lab-scale and semi-preparative scale, only the substances **1**–**9** were considered for the offline analysis. However, some fractions contained impurities, parts of which were tentatively identified to be minor compounds from the chamomile extract. These substances eluted especially at the beginning of the separation, i.e., the breakthrough of the mobile phase at a run time of about 10 min, which indicates that they are hardly soluble in the stationary phase (partition coefficient of approximately 0). Nevertheless, none of these compounds co-eluted with the target substances. At the end of each separation, the stationary phase was pumped out from the column and analyzed. It contained mainly compound **3** and traces of other sesquiterpenes (m/z 204). In general, nonpolar sesquiterpenes have a high affinity to the stationary phase (*n*-heptane/ethyl acetate-rich phase) and are strongly retained in the column.

A complete baseline separation of all chamomile sesquiterpenes and sesquiterpenoids was considered not feasible with the Arizona S system, because the P_i_ values of these compounds were very similar, i.e., the separation factor is too small. This expectation was confirmed by the separation experiments. Surprisingly, however, the isomers **8** and **9** were clearly separated by CCC and CPC, although co-elution of these two isomers had been expected from the initial solvent system screening. In contrast, the compounds **4** and **5** as well as **7** and **8** co-eluted, and were recovered as binary mixtures. Therefore, further isolation steps were applied to the respective fractions to obtain the pure compounds.

### Size-exclusion chromatography and silica gel column chromatography

Since we expected that the co-eluting substances possess similar polarities, SEC was chosen as a chromatographic technique which allows separation based on the molecular size. Methanol was successfully applied as eluent to achieve separation of **7** and **8** into two fractions, as confirmed by GC-MS measurements. However, although **8** and **9** were baseline separated by CPC, traces of **9** were present in the fractions of **8** after the SEC step. This is most likely due to isomeric conversion, since the concentration of **9** was also rising during storage (exclusion of light, −20 °C). Separation of **4** and **5** was achieved by consecutive column chromatography using silica gel as stationary phase and mixtures of *n*-hexane/ethyl acetate and toluene/methyl tert-butyl ether (MTBE) as mobile phase. Different solvents and binary mixtures were tested. The best solvent gradient for a separation was determined as a mixture of toluene and MTBE.

GC-MS and nuclear magnetic resonance (NMR) spectroscopy experiments were conducted to examine the purity of the isolated sesquiterpenoids spathulenol, α-bisabolol oxide B, α-bisabolone oxide A, α-bisabolol oxide A, and additionally of the compounds (*E*/*Z*)-en-yn-dicycloether. GC-MS data showed high purities of the target substances (**4** = 95%, **5** = 95%, **6** = 94%, **7** = 100%, **8** = 62%, **9** = 91%). NMR spectra were obtained from compounds **7** and **9** and were in good accordance with the literature [31–33]. The other compounds were not isolated in sufficient amounts to perform NMR.

## Conclusions

*Matricaria chamomilla* L. is an herbal plant rich in sesquiterpenes and sesquiterpenoids. The combination of SAFE and solid support-free liquid-liquid chromatography proved to be suitable to obtain several sesquiterpenoids in high purities (94–100%). The transfer of the separation method from a lab-scale CCC unit to the semi-preparative CPC unit was successful, and resulted, in combination with further consecutive separation steps, including silica gel column and size-exclusion chromatography, in the isolation of the target compounds spathulenol, α-bisabolol oxide B, α-bisabolone oxide A, α-bisabolol oxide A, and (*E*/*Z*)-en-yn-dicycloether. The here investigated approach is not only of interest with regard to industrial application due to its scalability. In this respect, it is likewise important to design cycle processes that guarantee optimized use of solvents and materials to ensure ecological compatibility. Accordingly, this work helps paving the path into the coming era of bioeconomy and valorization of plant-based bioactives, and their manifold potential beneficial effects. In this respect, we currently test the isolated sesquiterpenoids in dedicated physiological assays to elucidate their potential neurotropic modulatory activity, and to evaluate their chemosensory properties. The here described procedure for the isolation of natural compounds may be adapted in future work to isolate other bioactive compounds.

## Supplementary information

ESM 1 (PDF 138 kb).
