# Supplementary material for: Isolation of sesquiterpenoids from Matricaria chamomilla by means of solvent assisted flavor evaporation and centrifugal partition chromatography
Source: Anal Bioanal Chem. 2021 May 28;413(17):4387–96. doi: 10.1007/s00216-021-03400-w (PMC8245379; doi:10.1007/s00216-021-03400-w)
Supplement: Supplementary file 1 — (PDF 138 kb). [file 216_2021_3400_MOESM1_ESM.pdf]

Supplementary Information

Isolation of sesquiterpenoids from *Matricaria chamomilla* by means of solvent assisted flavor evaporation and centrifugal partition chromatography

Benedikt Slavik [1], Simon Roehrer [2], Helene M. Loos [1,3], Mirjana Minceva [2], Andrea Buettner [1,3]

[1] Chair of Aroma and Smell Research, Friedrich-Alexander-Universität Erlangen-Nürnberg (FAU), Henkestr. 9, 91054 Erlangen, Germany

[2] Biothermodynamics, TUM School of Life Sciences, Technical University of Munich, Maximus-von-Imhof-Forum 2, 85354 Freising, Germany

[3] Fraunhofer Institute for Process Engineering and Packaging IVV, Giggenhauser Straße 35, 85354 Freising, Germany

Authors to whom correspondence should be addressed:

Mirjana Minceva:

E-mail: mirjana.minceva@tum.de

Andrea Buettner:

E-mail: andrea.buettner@fau.de; andrea.buettner@ivv.fraunhofer.de

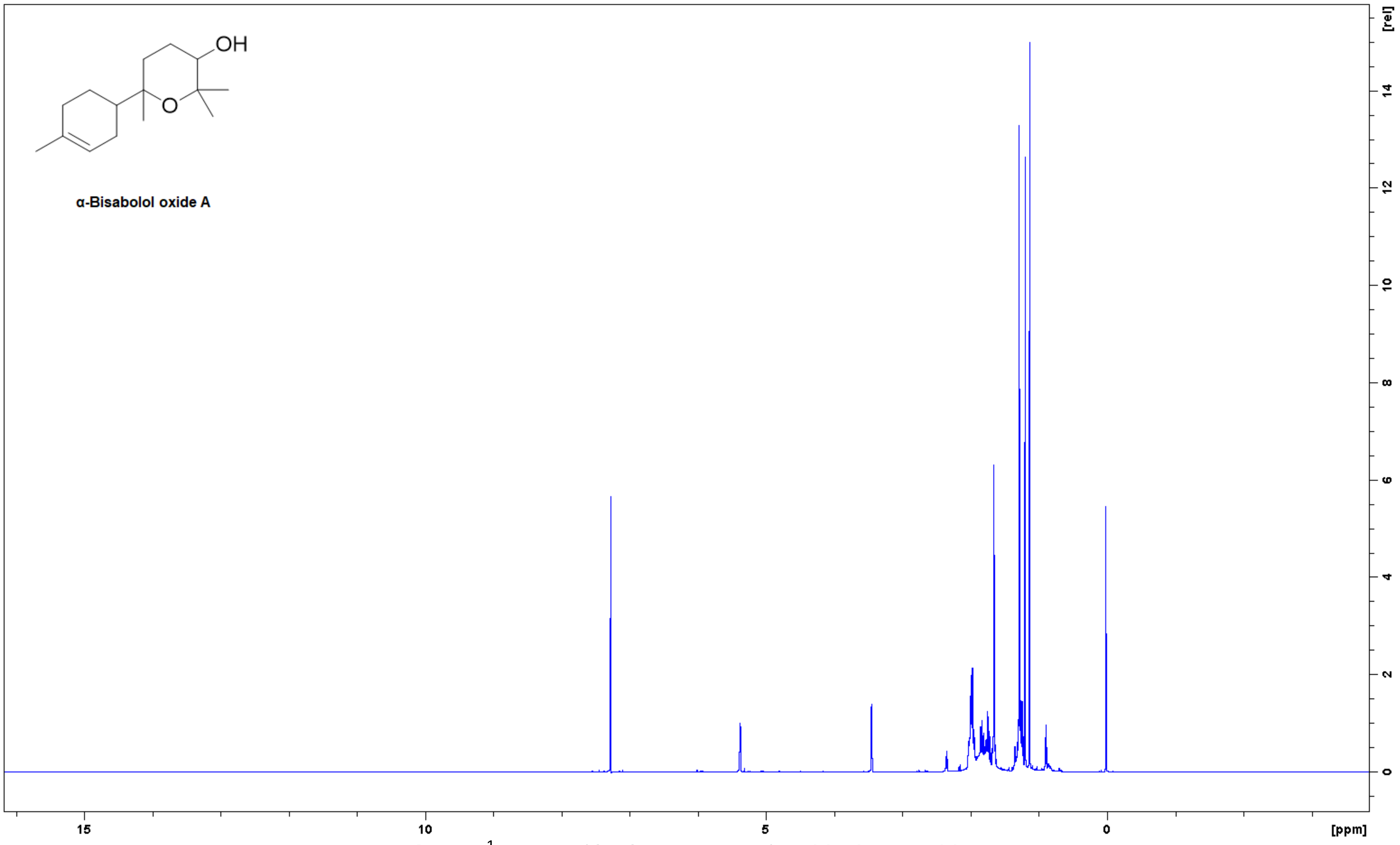

Fig. S1 ^{1}H NMR (CDCl3, 600 MHz), α-bisabolol oxide A.

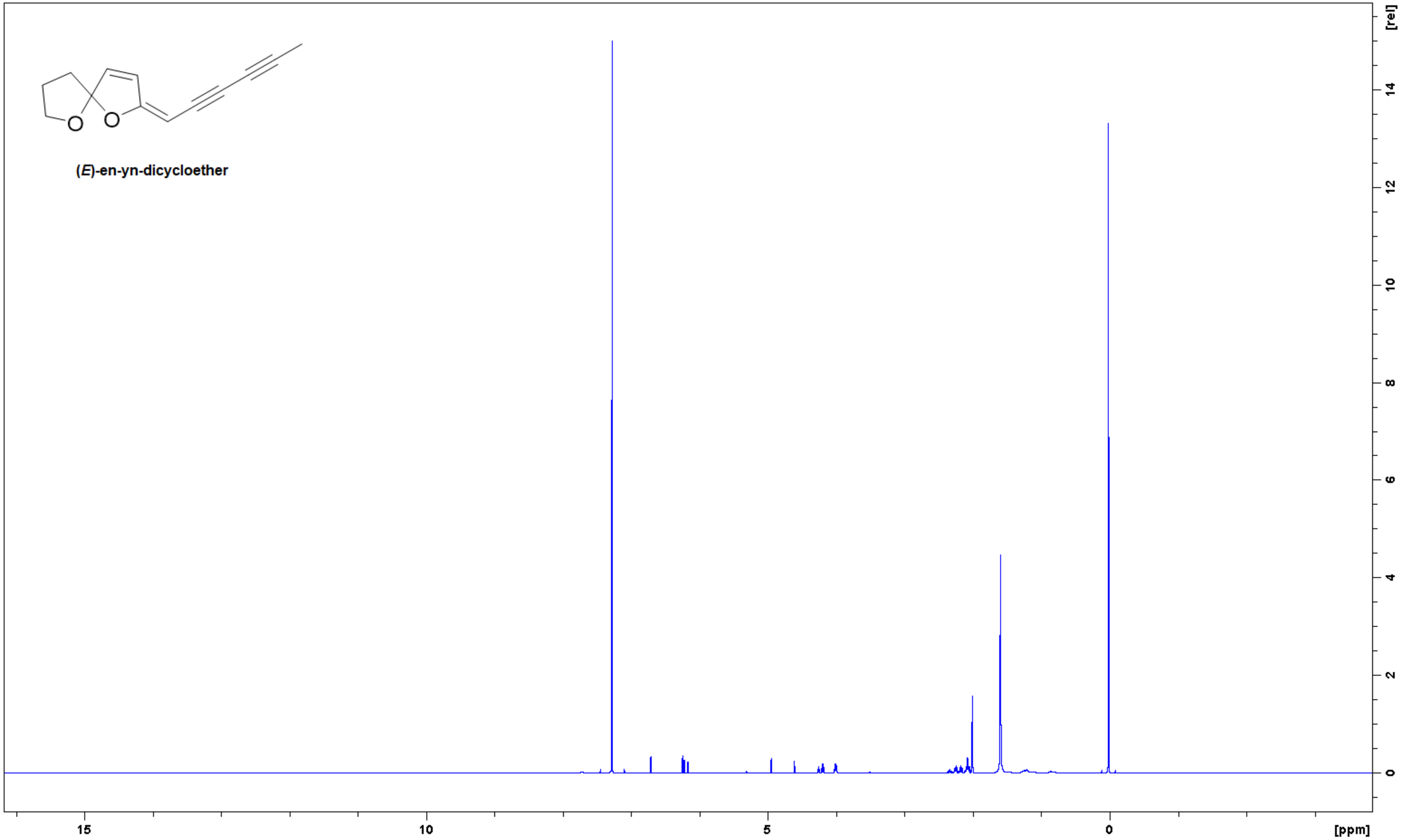

Fig. 2 ^{1}H NMR (CDCl3, 600 MHz), (*E*)-en-yn-dicycloether
